# An update of clinical issues from InSIGHT 2011

**DOI:** 10.1186/1897-4287-10-S2-A9

**Published:** 2012-04-12

**Authors:** L Lipton

**Affiliations:** 1 Ludwig Institute for Cancer Research, Royal Melbourne Hospital, Western Hospital, PO Box 2008, Royal Melbourne Hospital, Parkville 3050, Australia

## 

The fourth biennial InSIGHT meeting, held from 30th March to 2nd April 2011 in San Antonio Texas was a great success. The meeting was very well organized by hosts Dr Patrich Lynch and Dr Michael Rodriguez Bigas. Topics covered known genetic predispositions to gastrointestinal and other cancers, identification, risk stratification and surveillance and new developments in basic science.

